# Stabilizing Organic–Inorganic Lead Halide Perovskite Solar Cells With Efficiency Beyond 20%

**DOI:** 10.3389/fchem.2020.00592

**Published:** 2020-07-28

**Authors:** Ching Lin

**Affiliations:** Science & Technology Policy Research and Information Center, National Applied Research Laboratories, Taipei, Taiwan

**Keywords:** perovskite solar cells, stability, passivation, efficiency, defects, dopants, oriented crystal growth, charge transport materials

## Abstract

The power conversion efficiency (PCE) of organic–inorganic lead halide perovskite solar cells (PSCs) has exceeded 25%, approaching the best record of their silicon counterpart. However, lifetime issues still stand between PSCs and the goal of mass commercialization. For instance, most photoactive perovskites are hydrophilic, and moisture can quickly turn some of their constituents to compounds yielding trap states. Some perovskites are not thermally stable in the temperature window of solar cell operation and will transform into photo-inactive non-perovskites. If a perovskite is of an inadequate quality, e.g., vacancies, surface area, or grain boundaries per unit volume are high, there exist more defect sites, acting as migration pathways for perovskite ions under photo-bias, and the migration changes the perovskite's composition. An unstable perovskite/charge transport material (CTM) interface allows cross-contamination between molecules from both sides of the interface. Even without external stress, perovskite ions in an operating PSC undergo redox processes, which create defect states or initiate chemical chain reactions to accelerate PSC degradation. This mini-review discussed recent progress in solving issues, including the above, to stabilize PSCs with competitive PCE beyond 20%. The remarkable longevity of 15 PSCs under accelerated aging tests was probed in depth from three viewpoints: (1) perovskite compositions and dopants, (2) perovskite additives, and (3) CTMs. This mini-review, within which crucial perovskite-stabilizing methods were systematically analyzed, can be used as a quick-start guide when dealing with PSCs' stability in the future.

## Introduction

The power conversion efficiency (PCE) of organic–inorganic lead halide perovskite solar cells (PSCs) has exceeded 25%, approaching 26.7% of crystalline silicon solar cells (National Renewable Energy Laboratory, 2020). However, stability issues still keep PSCs from mass commercialization. A marketable solar cell requires a warranty of 20–25 years. One is considered to match this requirement if retaining ≥90% of initial PCE after an accelerated aging test (AAT) of a 1,000-h continuous operation under 100 mW cm^−2^ AM 1.5 G illumination (denoted as 90%@1,000 h here; Grancini et al., 2017). This mini-review briefed recent efforts on stabilizing PSCs to have PCEs beyond 20% and lifetimes that prove they are qualified or nearly qualified by 90%@1,000 h tests.

Organic–inorganic lead halide perovskite is obtained by reacting halides of lead and organic ammonium with dopants. Because the chemical bonds, including ionic bonds, hydrogen bonds, and van der Waals interactions, between the constituents are relatively weak, a perovskite can decompose under moisture, thermal, or light stress. The perovskite structure may undergo irreversible hydrolyzation when exposed to moisture (Song et al., 2016), and the hydrolyzation can be accelerated by heat (Han et al., 2015). Some perovskites even transform into photo-inactive non-perovskites in the temperature window of solar cell operation without moisture exposure (Li et al., 2016). Defect sites within a perovskite act as charge recombination centers and migration pathways for perovskite ions under photo-bias; the migration changes the perovskite's composition (Gao et al., 2020b). More degradation mechanisms are discussed in the corresponding sections.

Table 1 outlines device structures, champion PCEs, and AAT results and conditions of 15 highlighted PSCs (the abbreviations and chemical structures of the materials can be found in the Supplementary Material). They are ranked by lifetimes and named accordingly, while the ones surviving high-temperature AATs are prioritized. Although the PCE of D0 is far below 20%, its survival under 10,000-h AAT without any PCE loss makes it worth discussing. It should be noticed that AATs in these references are not standardized for commercial uses and alter from one another. An AAT temperature of ~60°C is close to that of a solar cell operating under the sun, whereas using a 25°C AAT may overestimate a PSC's performance. The column “lifetime” records residual percentages of the initial PCEs after the hours of AATs (for example, 95%@1,000 h represents 95% remained after 1,000-h aging). The AATs were all performed at the maximum power point (MPP tracking) except that for D10, which was exposed to continuous light under an open-circuit condition (open-circuit light-soaking). The MPP tracking ages a PSC more severely than the open-circuit light-soaking does. For example, D11 retained 92% of initial PCE after 1,500-h open-circuit light-soaking, but the PCE dropped to 91% of initial value only after 500-h MPP tracking. D0~D14's short-circuit photocurrent (*J*_SC_), open-circuit voltage (*V*_OC_), and fill factors (FFs) can be found in Supplementary Table 1. Considering the Shockley–Queisser (SQ) limit (theoretical PCE limit; see Park and Segawa, 2018) and using FFs of well-developed solar cells as references (Green et al., 2019), there are still ~20% for improvements in these aspects. Inefficient light harvest or incomplete carrier collection lowers a PSC's *J*_SC_, whereas electrical losses, such as carrier recombination and parasitic resistance, reduce *V*_OC_ or FFs (Jena et al., 2019). Although many reported PSCs with PCEs less than 20% had longer lifetimes, the insufficient PCEs kept them even farther from the goal of mass commercialization. Their PSC-stabilizing mechanisms may not be as effective when applied to a PSC with higher PCE. Scaling up the PSC area can worsen the problems further. Therefore, only D0 among them was discussed here as a representative example.

**Table 1:** Information about the highlighted 15 PSCs.

**Name**	**Anode/ETL/PVK/HTL/cathode**	**PCE (%)**	**Lifetime**	**Condition**	**Temp**.	**References**
D0	FTT/ZrO_2_^†^/(AVA)_2_PbI_4_/MAPbI_3_/C/G	11.9	>100%@10,000 h	Air, UV filtered, encap.	55	Grancini et al., 2017
D1	FTT/(Cs,FA,MA)Pb(I,Br)_3_^‡^/CuSCN/rGO/Au	20.4	95%@1,000 h	N_2_, unencap.	60	Arora et al., 2017
D2	FTT/SN, (FAI)_0.9_Cs_0.1_(PbI_2_)_1.05_/S/A	20.9	98.1%@1,000 h	Ar, unencap.	60	Bi et al., 2018
D3	ITO/SnO_2_/FA_x_MA_1−x_Pb_1+y_I_3_^‡^/Pb/Cl-GO/PTAA/Au	21.1	90%@1,000 h	Encap.	60	Wang Y. et al., 2019
D4	FTT/LiTFSI/(FAPbI_3_)_0.95_(MAPbBr_3_)_0.05_/HTAB-PVK/P3HT/Au	23.3	95%@1,370 h	RH30% air, encap.	25	Jung et al., 2019
D5	ITO/^S^C_60_/SnO_x_/PCBM/FA_0.83_MA_0.17_Pb_1.1_Br_0.50_I_2.80_/PDCBT/Ta-WO_x_/Au	21.2	95%@1,000 h	N_2_, unencap.	RT	Hou et al., 2017
D6	FTT/(Cs,FA,MA)PbI_3_^‡^/(FEAI)_2_PbI_4_/S/A/MgF_2_	22.2	90%@1,000 h	RH40–60% air, unencap.	RT	Liu et al., 2019
D7	ITO/SnO_2_/Rb_0.09_Cs_0.05_[(FA_0.85_MA_0.15_)Pb(I_0.85_Br_0.15_)_3_]/S/A	20.9	80%@1,000 h	Air, encap.	20	Ma et al., 2020
D8	FTT/SnO_2_/(Cs_0.17_FA_0.83_)Pb(I_0.82_Br_0.15_Cl_0.03_)_3_/S/A	20.5	80%@1,000 h	Ar, unencap.	RT	Gao et al., 2020a
D9	FTT/(Cs,FA,MA)Pb(I,Br)_3_^‡^/S/A	22.0	>100%@600 h	N_2_, unencap.	25	Seo et al., 2018
D10	ITO/SnO_2_/(FA,MA)Pb(I,Cl)_3_^‡^,^2D^ThMA/Spiro/MoO_3_/Au	21.5	94%@576 h	N_2_, unencap., open-circuit light-soaking	N/A	Zhou et al., 2019
D11	ITO/SnO_2_/Eu, (Cs,FA,MA)Pb(I,Br,Cl)_3_^‡^/PTAA,Spiro/Au	21.9	91%@500 h	N/A	N/A	Wang L. et al., 2019
D12	FTT/(FAPbI_3_)_0.95_(MAPbBr_3_)_0.05_/DM/Au	23.2	92.6%@310 h	RH25% air, encap.	25	Jeon et al., 2018
D13^*^	Cu/BCP/C_60_/Cs_0.05_FA_0.81_MA_0.14_PbI_2.55_Br_0.45_/PTAA/ITO	21.1	96.8%@1,200 h	RH60% air, encap.	65	Yang et al., 2019
D14^*^	Cu/BCP/C_60_/Cs_0.05_(FA_0.92_MA_0.08_)_0.95_Pb(I_0.92_Br_0.08_)_3_/PTAA/ITO	23.0	>100%@1,000 h	N_2_, UV filtered, encap.	40	Zheng et al., 2020

* *The only two p–i–ndevices discussed here*;

† *ZrO_2_spacer*;

‡ *the perovskite composition detail was not specified in the reference*.

**Figure 1:**
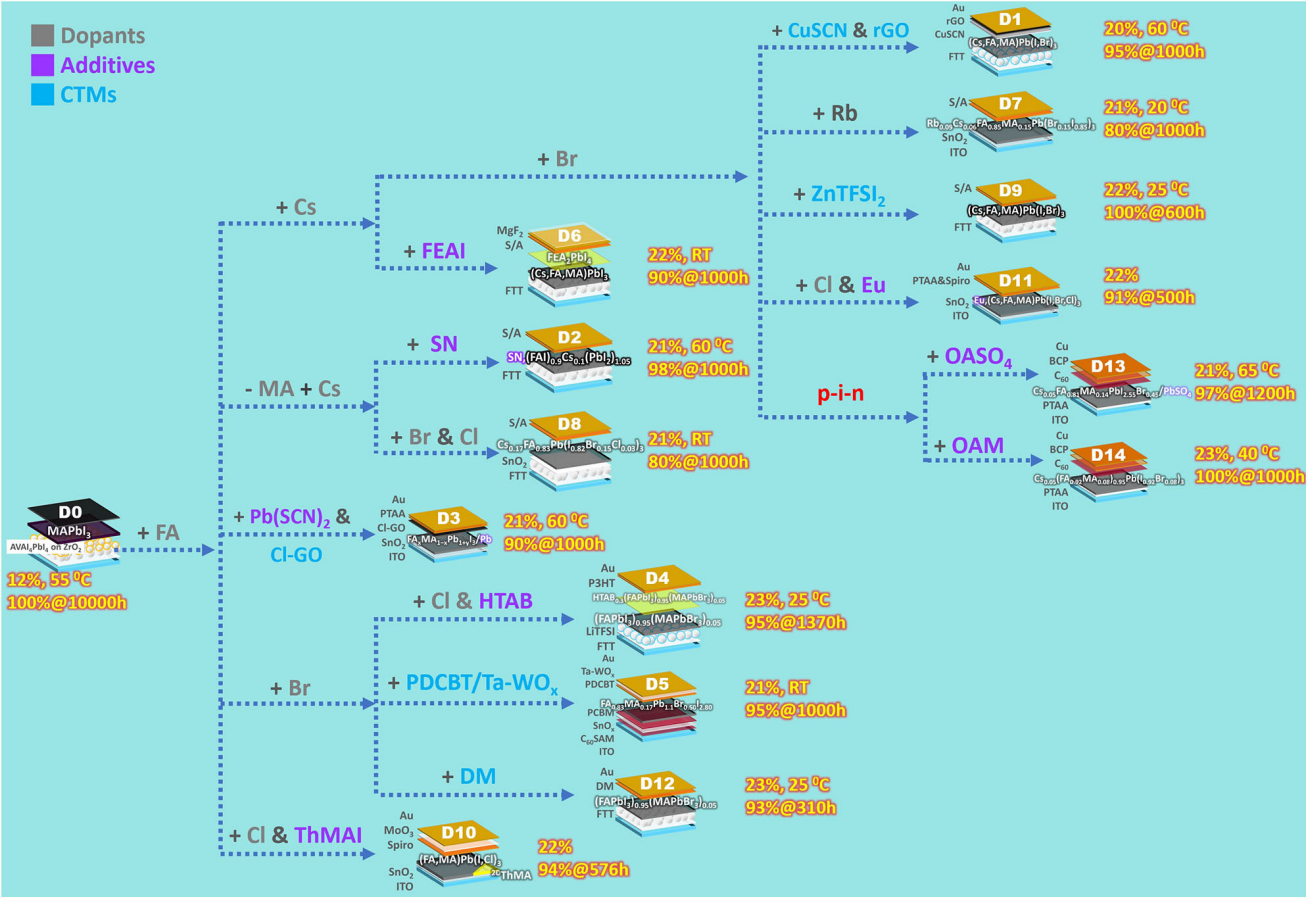
PSC-stabilizing flowchart according to key components applied to a PSC.

As summarized from the references, a PSC can be stabilized via (1) modifying perovskite composition by dopants, (2) applying perovskite additives, or (3) adjusting charge transport materials (CTMs). These three subjects are discussed in the following. D0~D14 are organized in Figure 1 according to dopants, additives, or CTMs applied to a PSC. The term dopant used here is defined as perovskite impurities altering a photoactive perovskite's intrinsic properties, while additives do not influence the photoactive perovskites' chemical composition *per se*, whose functions are discussed in the corresponding section.

## Perovskite Compositions and Dopants

A perovskite's decomposition normally starts at the surface. If the surface area per unit volume can be reduced, even MAPbI_3_, a perovskite considered very vulnerable, can have longevity. Grancini et al. (2017) reported a MAPbI_3_ PSC (D0) surviving a 10,000-h, 55°C AAT with zero loss in PCE. Slowly dried onto a mesoporous TiO_2_ ETM, the MAPbI_3_ film was formed bottom-up by a thin oriented phase within the mesoporous TiO_2_ and a tetragonal phase on top of it (the additive stimulating this process is discussed in the section of Perovskite Additives). The longevity of the MAPbI_3_ PSC is probably due solely to this ordered crystal rearrangement, which lowers the surface area per unit volume. Although the PCE of D0 stayed above the initial value throughout the AAT, it kept changing in the first 4,000 h. This phenomenon can be attributed to light- or field-induced ion migration, interfacial charge accumulation, etc., which is the evidence of MAPbI_3_'s instability.

The perovskite used in the first effective PSC was MAPbI_3_ (Kojima et al., 2009). Since then, the approach of incorporating dopants or totally replacing MA^+^ has been found to stabilize perovskites (Figure 1). The general formula of perovskite can be expressed as ABX_3_, wherein A is a cation (e.g., organic ammonium or other dopants), B is Pb^2+^, and X is a halogen anion. The Goldschmidt tolerance factor, determined by radii of A, B, and X, is an empirical index used to predict perovskites' structural stability (Li et al., 2016). An ideal photoactive perovskite has a cubic structure, within which ${\text{BX}}_{6}^{4-}$ forms a sub-lattice octahedral framework, while A^+^ ions occupy cube corners (Jena et al., 2019). Larger A^+^ ions usually result in a higher tolerance factor. For example, photoactive cubic (α)-phase FAPbI_3_ (effective ionic radius of FA^+^ is 2.53 Å; see Hoefler et al., 2017), having a large tolerance factor, is unstable at RT, and tends to convert to a photo-inactive non-perovskite hexagonal phase. When alloying FAPbI_3_ with small-tolerance-factor CsPbI_3_ (effective ionic radius of Cs^+^ is 1.88 Å), the cubic phase can be stabilized. Furthermore, Cs^+^ has strong interaction with the ${\text{BX}}_{6}^{4-}$ framework, which greatly improves the photostability (Chen et al., 2019). Incorporating small and oxidation-stable rubidium cation (effective ionic radius of Rb^+^ is 1.72 Å) stabilizes a perovskite in a similar manner.

On the other hand, incorporating X^−^ other than I^−^, namely, Cl^−^ and Br^−^, influences not merely the tolerance factor but the crystal growth, morphology, and hence the charge recombination. For example, the inclusion of MACl in perovskite precursor solution stabilizes FAPbI_3_'s cubic phase (Kim et al., 2019). Cl^−^ in this perovskite film were verified to be all evaporated after the following annealing (perovskite films for PSC use are annealed to enhance crystal growth), suggesting that Cl^−^ from precursor solutions may not exist in every annealed perovskite film (e.g., D4, D8, D10, and D11). The effects of dopants on PSCs' stability can be observed from D7 to D8, since their device structures are simpler, compared to the others in Table 1, without additives or fancy CTMs. The perovskites used in D7 and D8 are Rb_0.09_Cs_0.05_[(FA_0.85_MA_0.15_)Pb(I_0.85_Br_0.15_)_3_] and (Cs_0.17_FA_0.83_)Pb(I_0.82_Br_0.15_Cl_0.03_)_3_, respectively. Both coupling with the unstable HTM of spiro-OMeTAD (the instability is discussed in the corresponding section), D7 and D8 nonetheless retained >80% of initial PCEs after 1,000-h AATs. In addition, (Cs,FA)PbI_3_ is very thermally stable. The PSC with the device structure of FTT/(FAI)_0.9_Cs_0.1_(PbI_2_)_1.05_/S/A (the control compared to D2) retained 88.4% of its initial PCE after 1,000-h 60°C AATs.

## Perovskite Additives

The additives' summarized perovskite-stabilizing mechanisms are (i) crystallization promotion, (ii) surface passivation, and (iii) ion regeneration. A crystallization-promoting additive promotes a photoactive perovskite's crystal growth. A surface-passivating additive provokes the formation of a moisture barrier or passivates defects on a photoactive perovskite's surface. Both the crystallization-promoting and surface-passivating additives may induce a two-dimensional (2D) perovskite; these kinds of additives were defined according to the 2D perovskite's function of crystallization-promoting or surface-passivating. An ion-regenerating additive regenerates perovskite ions consumed after PSC operation. An additive may stabilize a perovskite via more than one mechanism.

### Crystallization Promotion

The first example of a crystallization-promoting molecule is AVAI, a 2D perovskite layer induced by which plays a foundation layer for photoactive 3D perovskite to grow orderly. The D0 device was made by infiltrating a perovskite precursor solution including 3 vol% AVAI through carbon cathode into a ZrO_2_ spacer layer on a nanoporous TiO_2_ ETM. After drying at 50°C, the precursor molecules self-assembled as a 2D/3D perovskite bilayer rather than mixed 2D/3D perovskites. A thin AVA_2_PbI_4_ 2D perovskite layer, with AVA's carboxylic acid group bonding the oxides, covered the ETM, and the 3D layer mentioned in the previous section grew on the 2D layer. The interface between the 2D and 3D perovskites oriented in a preferential growth direction of the 3D phase. Since the 2D layer substantially stayed close to the ETM, the AVAI additive contributed mainly to allow growing the stable, oriented 3D phase.

The second example is SN (see D2). An SN molecule provides three functional groups, –NH on the tetrazole moiety, thione (C=S), and ammonium. When filming a perovskite precursor solution containing SN molecules, they promote perovskite crystal growth via surface interactions, rather than incorporating into the perovskite structure. The surface interactions, including hydrogen-bonding the donor groups (–${\text{NH}}_{3}^{+}$, –NH) to the perovskite surface and coordinating thione to Pb^2+^, direct assembly pathways, suppress the formation of unwanted impurities such as PbI_2_, and stabilize the grown structure of α-phase FAPbI_3_. The D2 device retained 98.1% of initial PCE after 1,000-h 60°C AAT, within which SN molecules provided further improvement to the lifetime of already thermally stable (FAI)_0.9_Cs_0.1_(PbI_2_)_1.05_ PSCs, which retained aforementioned 88.4% of initial PCE.

The third example is ThMAI (see D10). A 2D/3D hybrid perovskite was formed by coating an organic ammonium halide solution containing ThMAI on top of a PbI_2_ film with subsequent annealing. In the 2D/3D hybrid perovskite film, ThMA cations' π-stacking thiophene rings self-assembled organic sheets, which spaced the 2D and 3D perovskites. ThMAI induced perovskite crystal growth perpendicular to the PSC substrate with highly oriented, enlarged crystal grains. The unencapsulated D10 device retained 94% of initial PCE after a 576-h continuous light illumination in N_2_ under open-circuit condition.

The fourth example is oleylamine (OAM; see D14). Filming a perovskite precursor solution including <0.3 mol% of OAM makes the filmed perovskite have OAMs assembled on grain surfaces. During perovskite growth, the OAM assemblies act like scaffolds restricting grains' tilt and enable the grains to grow perpendicularly to the substrate. In the meantime, OAMs are expelled to the enlarging grains' surfaces and passivate them. The oriented grains show anisotropic electronic properties, and the perovskite with OAM has lower trap-state density than that without OAM. The encapsulated D14 device survived 1,000-h 40°C AAT in N_2_ without any PCE decrease.

The fifth example is HTAB (see D4). HTAB, whose crystallization-promoted object is different from those of the others discussed above, directs HTM assembly. Contrary to AVAI, inducing a 2D perovskite layer close to the bottom ETM, HTAB induces an ultrathin wide-bandgap halide (WBH) layer in the topmost part of the original perovskite. P3HT was used as the HTM in D4. The WBH layer improves the self-assembling ability of P3HT while filming a P3HT solution onto the WBH layer. The part about P3HT is elucidated in the section of Charge Transport Materials.

### Surface Passivation

The aforementioned AVAI (D0), SN (D2), HTAB (D4), and ThMAI (D10) play a dual role in crystallization-promoting and surface-passivating. For D0, since the AVA_2_PbI_4_ 2D layer stays close to the TiO_2_ ETM, it may protect the photoactive 3D perovskite from TiO_2_, whose photocatalytic nature is the reason why it is chosen as an ETM in the first place. For D2, SN's ammonium group mitigates the “A” cation vacancies, and the thione group passivates coordinatively unsaturated Pb^2+^ on the crystal surface. For D4, the WBH layer improves the perovskite's resistivity to moisture by hydrophobic long carbon chains of HTA^+^. For D10, the bulky organic sheets probably suppress the perovskite's ions from migration.

The fifth example of perovskite-passivating molecules is FEAI (see D6). FEAI is a phenylethylammonium iodide (PEAI; see Lee et al., 2018) having its phenyl moiety perfluorinated. They both induced the formation of a 2D perovskite phase, i.e., (FEAI)_2_PbI_4_ phase by FEAI and (PEAI)_2_PbI_4_ phase by PEAI. Since the FEAI solution was coated upon a formed 3D perovskite layer and PEAI was coated as part of a perovskite precursor solution, (FEAI)_2_PbI_4_ crystals appeared uniformly within an 8-nm range on top of the corresponding 3D perovskite layer, while (PEAI)_2_PbI_4_ existed allover grain boundaries within its corresponding one. The unencapsulated D6 device with (FEAI)_2_PbI_4_ retained 90% of initial PCE after 1,000-h room-temperature AAT at MPP in RH40~60% air, while the encapsulated PSC with (PEAI)_2_PbI_4_, aged in a less harsh condition of open-circuit light-soaking, lost >20% of initial PCE after 500-h AAT. The (FEAI)_2_PbI_4_ and (PEAI)_2_PbI_4_ layers both protect 3D perovskites from moisture and inhibit ion migration, but the (FEAI)_2_PbI_4_ layer's better performance suggests ultra-hydrophobic fluorinated molecules' superiority. The (FEAI)_2_PbI_4_ 2D layer also mitigates charge collection losses between the incompatible hydrophilic perovskite and the hydrophobic HTM.

The sixth example is alkyl ammonium sulfate. A 3–4-nm wide-band-gap PbSO_4_ layer capping a perovskite's surface is induced by coating dilute octylammonium sulfate (OASO_4_ in Figure 1) solution onto a pristine perovskite film (see D13). A perovskite wrapped with a thicker one of this PbSO_4_ layer is even waterproof underwater. The encapsulated D13 device had enhanced *V*_OC_ and FFs, was hysteresis-free, and retained 96.8% of initial PCE after 1,200-h 65°C AAT in RH60% air.

### Ion Regeneration

During PSC operation, Pb^2+^ are reduced to Pb^0^, acting primary deep defect states, whereas I^0^ are oxidized from I^−^ and become carrier recombination centers. I^0^ further initiate chemical chain reactions to accelerate PSC degradation. Filming a perovskite precursor solution containing europium acetylacetonate [Eu(acac)_3_] creates Eu^3+^ ions in the filmed perovskite. When Eu^3+^ encounter Pb^0^, Eu^3+^ oxidize Pb^0^ and turn into Eu^2+^ themselves, which can reduce I^0^. The Eu^3+^/Eu^2+^ pairs can transfer electrons from Pb^0^ to I^0^ defects in a cyclic manner and regenerate Pb^2+^ and I^−^ during PSC operation. The device with the Eu^3+^/Eu^2+^ pairs retained 91% of initial PCE after 500-h AAT (see D11).

## Charge Transport Materials

Organic–inorganic lead halide perovskite's photoactivity was found when adopted as a solid-state dye for dye-sensitized solar cells (DSSC). The majority of PSC researchers have stuck with solid-state DSSC's n–i–p structure (Cappel et al., 2012), i.e., using FTO/c-TiO_2_/mp-TiO_2_ as the bottom anode–ETM combination and spiro-OMeTAD/Au as the capping HTM–cathode combination. In such a case, in order to avoid a leakage current, the spiro-OMeTAD layer should be thick enough (>100 nm) to conformally cover the perovskite layer. The conductivity of spiro-OMeTAD is not high enough to transport injected holes through the thick HTM, and dopants, i.e., bis(trifluoromethane)sulfonamide lithium (LiTFSI), 4-*tert*-butylpyridine (tBP), and cobalt(III) complexes, are included into the HTM to facilitate the hole transport. Incorporating dopants reduces glass transition temperature (*T*_g_) of spiro-OMeTAD to that below a solar cell's average operational temperature (~60°C). This causes PSC constituents (e.g., perovskite ions, HTM dopants, and electrode molecules) to migrate across the perovskite/HTM interface and deteriorates the PSC. Moreover, the ionic HTM dopants' hygroscopic nature brings degradation pathways to a PSC.

Increasing doped HTM's *T*_g_ eases this problem. When using DM as HTM (D12), which has higher *T*_g_ (~90°C) than that of spiro-OMeTAD (~50°C) as doped, the stability was improved but not satisfying (92.1%@310 h). In another study (D9), ZnTFSI_2_ was used to replace LiTFSI. ZnTFSI_2_, bulkier than LiTFSI, reduces doped spiro-OMeTAD's *T*_g_ less severely. The D9 device surprisingly had zero PCE loss after AAT at 25°C (>100%@600 h for D9 and 80%@600 h for PSC with LiTFSI). However, at a more realistic operational temperature of 50°C, the D9 device lost 20% of initial PCE after 100-h AAT. Using hygroscopic-ion-free HTM dopants is a better choice in this regard. Conductive tantalum-doped tungsten oxide (Ta-WO_x_), as a hygroscopic-ion-free dopant, enables the HTM of PDCBT to provide sufficient hole transport (D5). In addition, the deeper highest occupied molecular orbital (HOMO) level of PDCBT (−5.3 eV) than that of spiro-OMeTAD (−5.0 eV) inhibits reaction between the HTM and I^0^ from the perovskite when photo-biased. The unencapsulated D5 device retained 95% of initial PCE after 1,000-h room-temperature AAT in N_2_.

Eliminating dopants is an obvious solution if the HTM has sufficient hole mobility. P3HT's acceptable energy level and hole mobility as high as 0.1 cm^2^ V^−1^ s^−1^ without any dopants make it a superior HTM candidate. However, the high mobility can be achieved only when P3HT molecules assemble orderly. HTA^+^ ions in the aforementioned WBH layer (D4) offer n-hexyl moieties to interdigitate with P3HT molecules' n-hexyl moieties and enable them to orderly assemble fibril structures. The encapsulated D4 device retained 95% of initial PCE after 1,370-h 25°C AAT in RH30% air. Another solution is inserting a spacer between perovskite and HTM to inhibit constituent cross-contamination. Yet, a spacer interferes in hole transport. A Pb-rich layer was induced onto the perovskite film of D3 by coating Pb(SCN)_2_ solution, ensuring stable bonding between the film and the spacer, i.e., Pb–O and Pb–Cl between the film and the spacer of chlorinated graphene oxide, and hence sufficient hole transport. Despite coupling with doped PTAA, whose *T*_g_ is lower than that of doped spiro-OMeTAD, the encapsulated D3 device retained 90% of initial PCE after 1,000-h 60°C ATT. Using an undoped, morphologically stable inorganic HTM is an even better strategy. CuSCN, a cheap p-type semiconductor, has a work function well-aligned with the perovskites', high hole mobility, and excellent thermal stability (Zhao et al., 2015). The problem of intermixing with hydrophilic perovskites when solution-processed had kept CuSCN from being effective HTM for n–i–p PSCs. Quickly coating CuSCN solution onto a spinning perovskite film enables the formed n–i–p PSC to have a conformal CuSCN HTM. After solving the issue of CuSCN HTM's reacting with Au cathode under photo-bias by inserting a reduced graphene oxide spacer between the HTM and the cathode, the unencapsulated device (D1) retained 95% of initial PCE after 1,000-h 60°C ATT in N_2_. A similar n–i–p PSC applying CuSCN/cheap carbon paste as the HTM-cathode combination (Arora et al., 2019) further had extreme longevity (95%@2,000 h), though with a slightly lower PCE of 18% than the target ≥20% of this mini-review.

So far, the PSCs discussed in this section are n–i–p PSCs. n–i–p PSCs suffer from the problems above, and their most used anode-ETM combination, i.e., FTO/c-TiO_2_/mp-TiO_2_, involving high-temperature processes such as spray pyrolysis, is difficult to fabricate. The easy-to-fabricate p–i–n device structure of cathode/HTM/perovskite/C_60_/BCP/anode was proven effective for PSCs (Liu et al., 2018). The ETM of 1 nm C_60_ with 7.5 nm BCP is thick enough to protect the device from a current leak, and the HTM can be so thin (~10 nm) that dopants are no more needed. With the perovskite additives discussed before (D13 and D14), promising stability of p–i–n PSCs was achieved.

## Conclusion

This mini-review summarized recent progress in stabilizing PSCs with PCE beyond 20%. An ideal long-lived PSC should have stable compositions of the perovskite and CTMs under moisture, thermal, or light stress. Doping a perovskite can manipulate its tolerance factor and elevate its structural and thermal stabilities to a preliminary level (e.g., D7 and D8). A perovskite's surface has a higher defect density than that of its bulk, which makes the surface more sensitive to the stresses. The surface area can be diminished by improving the perovskite's crystallinity employing crystallization-promoting additives (e.g., D0, D2, D10, and D14). Applying a surface-passivating additive reduces the surface defect density (e.g., D0, D2, D4, D6, D10, and D13); if the surface-passivating additive induces the formation of a hydrophobic moisture barrier, the perovskite can further resist the moisture stress (e.g., D4, D6, and D13). An ion-regenerating additive can resume perovskite ions consumed after PSC operation (e.g., D11). Regarding CTM stability, HTM's moisture stability can be improved by reducing HTM's hygroscopicity (e.g., D4 and D5). The thermal stability can be enhanced by applying HTMs with higher or without *T*_g_ (e.g., D1, D4, D9, and D12). Eliminating sensitive perovskite/HTM interface (e.g., D3) or adopting a stable p–i–n structure (e.g., D13 and D14) solves both moisture and thermal problems.

Promising dopants, additives, and CTMs of D0–D14, like their predecessors, will soon be used as standard components of a PSC. For example, the easy-to-fabricate p–i–n device structure may be applied with a perovskite precursor solution including ratio-optimized dopants and a crystallization-promoting additive like SN filmed to increase the crystallinity; an OASO_4_ solution may be coated onto the pristine perovskite film to enhance its moisture resistivity. Although the results were encouraging, to more explicitly reveal a PSC's realistic performance, future researchers are suggested to use an AAT condition with unencapsulated PSC aged under appropriate, controlled humidity and temperature. For high-efficiency PSCs surviving high-temperature AATs have been getting more common, the next challenge will be large-area PSC mass production.

## Author Contributions

The author confirms being the sole contributor of this work and has approved it for publication.

## Conflict of Interest

The author declares that the research was conducted in the absence of any commercial or financial relationships that could be construed as a potential conflict of interest.
