# Supplementary material for: Stabilizing Organic–Inorganic Lead Halide Perovskite Solar Cells With Efficiency Beyond 20%
Source: Front Chem. 2020 Jul 28;8:592. doi: 10.3389/fchem.2020.00592 (PMC7399487; doi:10.3389/fchem.2020.00592)
Supplement: Supplementary file 1 [file Table_1.pdf]

Supplementary Material

Supplementary Text

In Table 1 of the mini-review, ETM is electron transport material, PVK perovskite, and HTM hole transport material. FTT (FTO/c-TiO_2/mp-TiO_2) represents the most used anode-ETM combination, namely mesoporous TiO_2 on compact-TiO_2-coated fluorine doped tin oxide. ITO is indium tin oxide. Structures of chemicals in the following can be found in the **Supplementary Figures 1-3**. BCP is bathocuproine. $^{s}C_{60}$ represents a self-assembling C_{60} molecule. PCBM is phenyl-C61-butyric acid methyl ester. MA is methylammonium cation, and FA is formamidinium cation. AVA, SN, HTAB, FEAI, and ThMA represent aminovaleric acid, 3-(5-mercapto-1*H*-tetrazol-1-yl)benzenaminium iodide, n-hexyl trimethyl ammonium bromide, pentafluorophenylethylammonium iodide, 2-thiophenemethylammonium iodide. HTAB-PVK is $HTAB_{0.3}(FAPbI_3)_{0.95}(MAPbBr_3)_{0.05}$, a wide-bandgap halide (WBH), while ^{2D}ThMA is $(ThMA)_2PbI_4$, a 2D perovskite. C/G is carbon black/graphite composite slurry. rGO is reduced graphene oxide, and Cl-GO is chlorinated graphene oxide. PTAA is poly(triaryl amine), P3HT is poly(3-hexylthiophene-2,5-diyl), and PDCBT is poly[5,5′-bis(2-butyloctyl)-(2,2′-bithiophene)-4,4′-dicarboxylate-alt-5,5′-2,2′-bithiophene]. DM is $N^2,N^{2'},N^7,N^{7'}$-Tetrakis(9,9-dimethyl-9H-fluoren-2-yl)-$N^2,N^{2'},N^7,N^{7'}$tetrakis(4-methoxyphenyl)-9,9′-spirobi[fluorene]-2,2′,7,7′-tetraamine. Spiro represents $N^2,N^2,N^{2'},N^{2'},N^7,N^7,N^{7'},N^{7'}$-Octakis(4-methoxyphenyl)- 9,9′-spirobi[9*H*-fluorene]-2,2′,7,7′-tetramine (spiro-OMeTAD or s-OMeTAD), while S/A represents the most used HTL-cathode combination, namely spiro-OMeTAD-gold (spiro-OMeTAD/Au).

Supplementary Table 1

Name	Anode/ETL / PVK / HTL/Cathode	J_{SC} (mA cm^{-2})	V_{OC} (V)	FF (%)	PCE (%)	Reference
D0	FTT/ZrO_2 / $(AVA)_2PbI_4/MAPbI_3$ / C/G	23.6	0.86	58.7	11.9	Grancini et al., 2017
D1	FTT / $(Cs,FA,MA)Pb(I,Br)_3$ / CuSCN/rGO/Au	23.2	1.11	78.2	20.4	Arora et al., 2017
D2	FTT / $(FAI)_{0.9}Cs_{0.1}(PbI_2)_{1.05}$/SN / S/A	24.0	1.15	75.0	20.9	Bi et al., 2018
D3	ITO/SnO_2 / $FA_xMA_{1-x}Pb_{1+y}I_3$/Pb / Cl-GO/PTAA/Au	23.8	1.12	79.0	21.1	Wang et al., 2019b
D4	FTT/LiTFSI / $(FAPbI_3)_{0.95}(MAPbBr_3Cl_0)_{0.05}$/HTAB-PVK / P3HT/Au	24.9	1.15	81.4	23.3	Jung et al., 2019
D5	ITO/$^{S}C_{60}$/SnO_x/PCBM / $FA_{0.83}MA_{0.17}Pb_{1.1}Br_{0.50}I_{2.80}$ / PDCBT/Ta-WO_x/Au	22.7	1.17	80.0	21.2	Hou et al., 2017
D6	FTT / $(Cs,FA,MA)PbI_3/(FEAI)_2PbI_4$ / S/A/MgF_2	25.8	1.10	78.4	22.2	Liu et al., 2019
D7	ITO/SnO_2 / $Rb_{0.09}Cs_{0.05}[(FA_{0.85}MA_{0.15})Pb(I_{0.85}Br_{0.15})_3]$ / S/A	24.0	1.16	75.9	20.9	Ma et al., 2020
D8	FTT/SnO_2 / $(Cs_{0.17}FA_{0.83})Pb(I_{0.82}Br_{0.15}Cl_{0.03})_3$ / S/A	23.3	1.12	78.3	20.5	Gao et al., 2020a
D9	FTT / $(Cs,FA,MA)Pb(I,Br)_3$ / S/A	23.8	1.16	78.8	22.0	Seo et al., 2018
D10	ITO/SnO_2 / $(FA,MA)Pb(I,Cl)_3$,^{2D}ThMA / Spiro/MoO_3/Au	22.8	1.16	81.0	21.5	Zhou et al., 2019
D11	ITO/SnO_2 / $(Cs,FA,MA)Pb(I,Br,Cl)_3$ / PTAA,Spiro/S/A	23.5	1.15	80.7	21.9	Wang et al., 2019a
D12	FTT / $(FAPbI_3)_{0.95}(MAPbBr_3)_{0.05}$ / DM/Au	24.9	1.14	81.0	23.2	Jeon et al., 2018
D13	Cu/BCP/C_{60} / $Cs_{0.05}FA_{0.81}MA_{0.14}PbI_{2.55}Br_{0.45}$ / PTAA/ITO	22.6	1.16	80.4	21.1	Yang et al., 2019
D14	Cu/BCP/C_{60} / $Cs_{0.05}(FA_{0.92}MA_{0.08})_{0.95}Pb(I_{0.92}Br_{0.08})_3$ / PTAA/ITO	24.1	1.17	81.6	23.0	Zheng et al., 2020

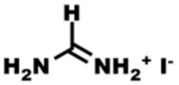

Formamidinium iodide (FAI)

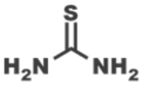

Thiourea

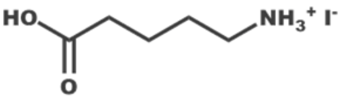

aminovaleric acid iodide (AVAI)

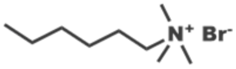

n-Hexyl trimethyl ammonium bromide (HTAB)

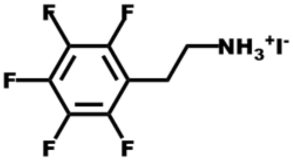

Pentafluorophenylethylammonium iodide (FEAI)

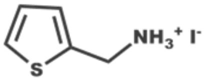

2-Thiophenemethylammonium iodide (ThMAI)

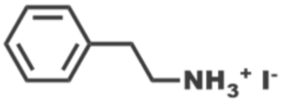

Phenethylammonium iodide (PEAI)

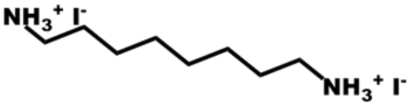

1,8-octanediammonium iodide (ODAI)

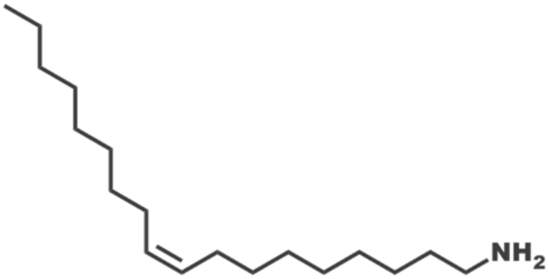

oleylamine (OAM)

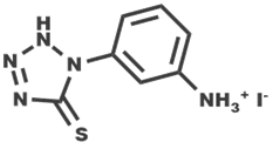

3-(5-mercapto-1*H*-tetrazol-1-yl)benzenaminium iodide (SN)

Supplementary Figure 1. Perovskite dopants or additives discussed in the mini-review.

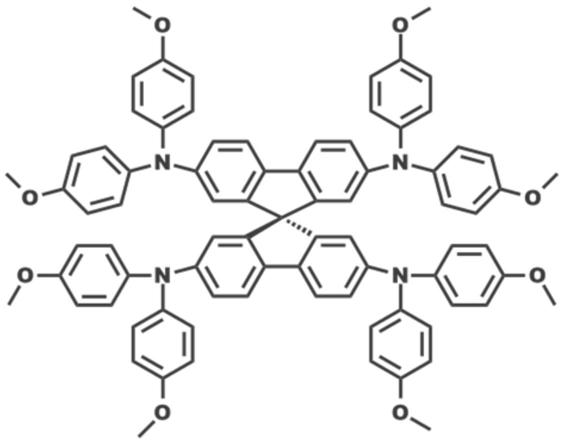

$N^{2},N^{2},N^{2'},N^{2'},N^{7},N^{7},N^{7'},N^{7'}$-Octakis(4-methoxyphenyl)- 9,9′-spirobi[9*H*-fluorene]-2,2′,7,7′-tetramine
(spiro-OMeTAD)

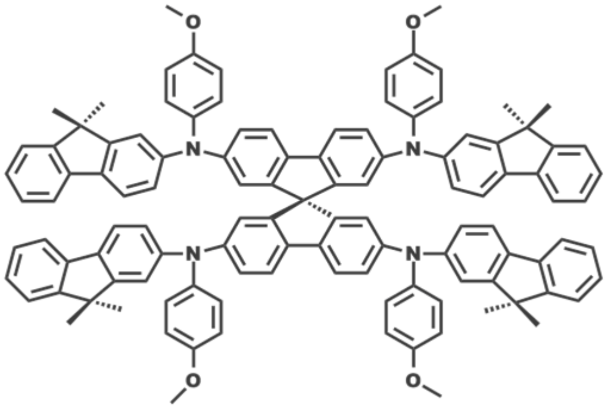

$N^{2},N^{2'},N^{7},N^{7'}$-Tetrakis(9,9-dimethyl-9H-fluoren-2-yl)-$N^{2},N^{2'},N^{7},N^{7'}$tetrakis(4-methoxyphenyl)-9,9′- spirobi[fluorene]-2,2′,7,7′- tetraamine (DM)

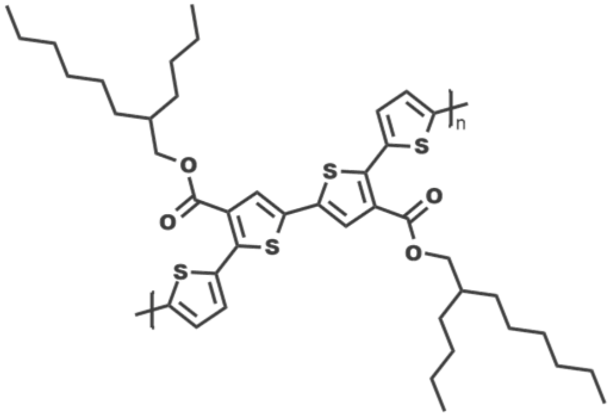

Poly[5,5′-bis(2-butyloctyl)-(2,2′-bithiophene)-4,4′-dicarboxylate-alt-5,5′-2,2′-bithiophene] (PDCBT)

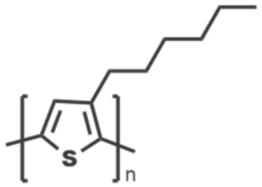

Poly(3-hexylthiophene-2,5-diyl) (P3HT)

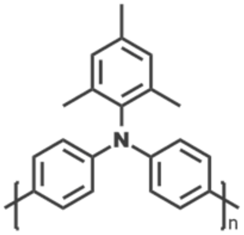

Poly(triaryl amine) (PTAA)

Supplementary Figure 2. Hole transport materials discussed in the mini-review.

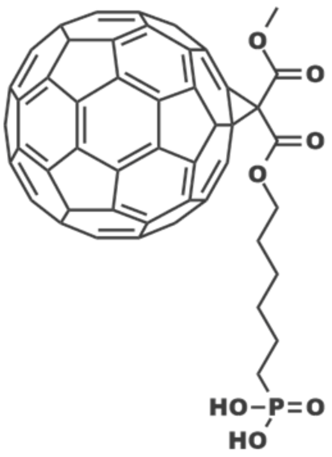

$^{S}C_{60}$: C_{60}SAM

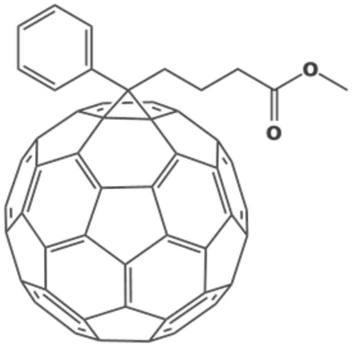

Phenyl-C61-butyric acid methyl ester (PCBM)

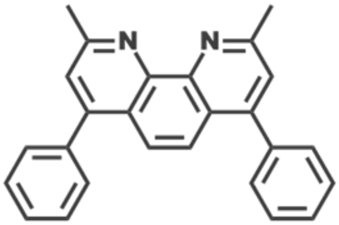

Bathocuproine (BCP)

Supplementary Figure 3. Electron transport materials discussed in the mini-review.
